# Community Engagement, Jurisdictional Experience, and Previous Tobacco-Related Ordinances in Neighboring Communities as Drivers of Flavored Tobacco Bans in Los Angeles County

**DOI:** 10.5888/pcd21.230284

**Published:** 2024-05-02

**Authors:** Dana Guglielmo, Andy Dang, Lori Fischbach, Ruth Toruno, Gladis Chavez-Sosa, Montgomery Messex, Tonya Gorham Gallow, Claud Moradian, Tony Kuo

**Affiliations:** 1 Tobacco Control and Prevention Program, Division of Chronic Disease and Injury Prevention, Los Angeles County Department of Public Health, Los Angeles, California; 2 Research and Evaluation, Division of Chronic Disease and Injury Prevention, Los Angeles County Department of Public Health, Los Angeles, California; 3 Department of Family Medicine, David Geffen School of Medicine, University of California, Los Angeles; 4 Department of Epidemiology, Fielding School of Public Health, University of California, Los Angeles; 5 Population Health Program, Clinical and Translational Science Institute, University of California, Los Angeles

## Abstract

We examined whether a community engagement approach and jurisdictional attributes were associated with local action to restrict the sale of flavored tobacco products in Los Angeles County during 2019–2022. We estimated crude and adjusted risk ratios to examine these associations. Jurisdictions that used an active community engagement approach to adopt a flavored tobacco ban ordinance, those with previous experience adopting other tobacco-related ordinances, and those located next to communities that have an existing tobacco retail license ordinance were more likely than jurisdictions without these attributes to adopt a new ordinance to restrict the sale of flavored tobacco products. Efforts to adopt such an ordinance were generally more successful in jurisdictions where community members were engaged and policy makers were familiar with the adoption of public health ordinances.

**Summary**

**What is already known on this topic?**

Local ordinances that restrict the sale of flavored tobacco products can markedly decrease their use. However, the prerequisite conditions and processes needed to advance the adoption of such ordinances are not well understood.

**What is added by this report?**

This study provides data supporting use of a community engagement approach, centered on the adoption and strengthening of tobacco retail license ordinances that restrict or ban the sale of flavored tobacco products, to counter the harmful effects of vaping and flavored tobacco use.

**What are the implications for public health practice?**

A community engagement approach that uses a policy adoption campaign can facilitate public support for ordinances that restrict the sale of flavored tobacco products.

## Objective

Use of flavored tobacco is a national epidemic (1,2). To expand its customer base, the tobacco industry has aggressively marketed these products to young people and other vulnerable groups, such as women of reproductive age and racial and ethnic minority populations (3).

Local ordinances that restrict the sale of flavored tobacco products (also known as flavored tobacco bans) represent an effective strategy to decrease the use of these products (4). Factors associated with adopting local tobacco-related ordinances may include a large population size, a relatively low prevalence of smoking, voting history, a higher income or education level, and geographic clustering (5–7). However, the conditions and processes that affect adoption of local flavored tobacco bans are not well understood. To address this gap in knowledge, this study in Los Angeles County, California, sought to assess 1) whether a community engagement campaign centered on adopting an ordinance to restrict flavored tobacco products could drive local jurisdictions to act and 2) whether other jurisdictional attributes affect decisions to adopt such an ordinance.

## Methods

The Tobacco Control and Prevention Program in the Los Angeles County Department of Public Health (LACDPH) conducted an analysis of a community engagement approach used by several jurisdictions to adopt a flavored tobacco ban ordinance in their communities. This approach included the following phases: 1) community assessment, 2) campaign strategy development, 3) coalition building, 4) campaign implementation, and 5) policy (ordinance) adoption (8). From 2019 through 2022, local community-based organizations used this approach (hereinafter, a “flavored tobacco ban campaign”) to help municipalities and the County of Los Angeles government adopt tobacco retail license ordinances that restrict the sale of flavored tobacco products. A tobacco retail license ordinance is a jurisdiction-specific policy that sets forth requirements and conditions that retailers need to meet in order to sell any tobacco product within a regulated region. For this effort, the Tobacco Control and Prevention Program selected 20 jurisdictions to conduct flavored tobacco ban campaigns throughout Los Angeles County. 

Los Angeles County covers more than 4,700 square miles, comprising 89 jurisdictions (88 cities and 1 large unincorporated area, which is not part of any city). Of these jurisdictions, 3 cities had previously adopted a flavored tobacco ban ordinance. Our study focused on the remaining 86 jurisdictions.

In 2018, the prevalence of menthol cigarette use in Los Angeles County was 4.8% (LACDPH, unpublished data, 2018). The prevalence of other flavored tobacco use was 4.2% overall and 12.7% among adults aged 18 to 24 years. By jurisdiction, the prevalence ranged from 5% to 18% for cigarette smoking and 4% to 11% for vaping (LACDPH, unpublished data, 2018).

We collected data on jurisdictions that implemented a flavored tobacco ban campaign and/or adopted a flavored tobacco ban ordinance, and the following jurisdictional attributes that may affect adoption: population size, geographic region (jurisdictions were grouped into 10 regions), number of neighboring communities with an existing tobacco retail license ordinance, sociodemographic characteristics (age, race and ethnicity, education), number of tobacco retailers, previous adoption of other tobacco-related ordinances (eg, for multiunit housing, outdoor spaces), other concurrent tobacco-related campaigns for multiunit housing or outdoor spaces, revenue per capita, and prevalence of tobacco product use. These data came from the following sources: 1) the Tobacco Control and Prevention Program ordinance tracking database (unpublished data, 2023), 2) the Los Angeles County Health Survey (unpublished data, LACDPH, 2018), 3) the US Census (9), and 4) the California Tobacco Health Assessment Tool (10). We used Fisher exact tests, Satterthwaite *t* tests, and Kruskal–Wallis tests to compare jurisdictions that implemented a flavored tobacco ban campaign and jurisdictions that did not. To assess the effect of a flavored tobacco ban campaign on the adoption of a flavored tobacco ban ordinance, we conducted a modified Poisson regression using SAS version 9.4 (SAS Institute Inc) (11), which estimated crude risk ratios and adjusted risk ratios while controlling for confounders. We performed similar analyses to assess the effect of jurisdictional attributes on the adoption of a flavored tobacco ban ordinance. We used ArcGIS version 10.8 (Esri) to create a thematic map of Los Angeles County showing jurisdictions that implemented a flavored tobacco ban campaign and adopted a flavored ban ordinance over time.

## Results

Overall, we did not observe any differences in sociodemographic characteristics among the 20 jurisdictions that implemented a flavored tobacco ban campaign versus the 66 jurisdictions that did not (Table 1). We did, however, observe differences in previous adoption of tobacco-related ordinances for jurisdictions with versus jurisdictions without a flavored tobacco ban campaign. For example, 8 of 20 jurisdictions (40.0%) with a flavored tobacco ban campaign had previously (before 2019) adopted an ordinance restricting smoking in multiunit housing, whereas only 4 of 66 jurisdictions (6.1%) without a flavored tobacco ban campaign had adopted such an ordinance. Similarly, 16 of 20 jurisdictions (80.0%) with a flavored tobacco ban campaign had adopted any tobacco retail license ordinance before 2019, whereas only 27 of 66 jurisdictions (40.9%) without a flavored tobacco ban campaign had adopted such an ordinance.

**Table 1:** Attributes of the 86 Jurisdictions^a^ Included in Study Analyses, by Implementation of a Flavored Tobacco Ban Campaign, Los Angeles County, 2019–2021^b^

Attribute	Flavored Tobacco Ban Campaign		*P* value
	Yes (n = 20)	No (n = 66)	
**Population size, median (range)**	61,873 (17,243−3,902,440)	39,931 (244−466,565)	.24^c^
**Geographic region, no. (%)**			
Central Los Angeles	1 (5.0)	1 (1.5)	.41^d^
Northwest/Antelope Valley	1 (5.0)	3 (4.6)	>.99^d^
San Gabriel Valley	6 (30.0)	22 (33.3)	>.99^d^
San Fernando Valley	4 (20.0)	3 (4.6)	.05^d^
Pomona Valley	2 (10.0)	2 (3.0)	.23^d^
Southeast	8 (40.0)	14 (21.2)	.14^d^
Harbor	2 (10.0)	5 (7.6)	.66^d^
South Bay	5 (25.0)	10 (15.2)	.33^c^
Westside	3 (15.0)	1 (1.5)	.04^d^
Santa Monica Mountains	1 (1.5)	5 (7.6)	>.99^d^
**Socioeconomic**			
Revenue per capita, median (range), $	1,608 (443−7,155)	1,302 (467−1,295,313)	.47^c^
Annual household income, median (range), $	74,494 (54,535−126,683)	86,378 (50,311−2,500,015)	.54^c^
Households below the federal poverty level, mean % (95% CI)	11.6 (9.5–13.7)	10.4 (9.1–11.7)	.32^e^
**No. of tobacco retailers, median (range)**	53.5 (10−3,469)	30.5 (0−409)	.20^c^
**Age, mean % (95% CI)**			
<21 y	25.7 (24.0–27.4)	25.7 (24.4–27.0)	.99^e^
21–60 y	53.5 (52.1–55.0)	51.6 (50.2–53.1)	.06^e^
>60 y	20.8 (18.5–23.0)	22.7 (21.1–24.2)	.16^e^
**Race and ethnicity, mean % (95% CI)**			
Asian/Pacific Islander	19.4 (10.9–27.8)	18.5 (13.9–23.0)	.85^e^
Black	7.3 (2.5–12.0)	4.4 (3.1–5.7)	.23^e^
Hispanic	47.6 (35.6–59.7)	43.8 (36.6–51.1)	.58^e^
White	22.4 (12.5–32.4)	29.7 (23.8–35.7)	.20^e^
Other	3.3 (2.2–4.4)	3.7 (3.1–4.3)	.85^e^
**Highest level of education attained, mean % (95% CI)**			
Less than Grade 9	11.8 (7.8–16.0)	9.3 (7.2–16.0)	.28^e^
Grade 9–11	7.7 (5.5–9.9)	7.0 (5.8–8.3)	.57^e^
High school diploma	21.4 (18.2–24.6)	19.2 (17.2–21.1)	.24^e^
Some college	19.0 (17.1–20.8)	18.6 (17.5–19.8)	.76^e^
Associate degree	7.2 (6.3–8.1)	7.4 (6.6–8.1)	.78^e^
Bachelor’s degree	21.4 (16.5–26.3)	23.8 (20.9–26.7)	.39^e^
Graduate or professional degree	11.6 (7.4–15.8)	14.7 (12.0–17.5)	.20^e^
**Tobacco use, mean % (95% CI)**			
Current smoker	10.7 (9.5–11.9)	9.9 (9.3–10.6)	.23^e^
Current e-cigarette user	6.5 (5.9–7.2)	6.9 (6.6–7.2)	.31^e^
Ever e-cigarette user	12.7 (11.8–13.6)	13.0 (12.6–13.5)	.53^e^
**Tobacco-related ordinance or policy campaign, no. (%)**			
Adoption of tobacco-related ordinance for outdoor areas before 2019	19 (95.0)	46 (69.7)	.02^d^
Adoption of tobacco-related ordinance for multiunit housing before 2019	8 (40.0)	4 (6.1)	<.001^d^
Adoption of any TRL ordinance before 2019	16 (80.0)	27 (40.9)	.004^d^
Adoption of any tobacco-related ordinance before 2019	19 (95.0)	48 (72.7)	.06^d^
Other concurrent tobacco-related campaign	4 (20.0)	47 (71.2)	<.001^d^
**Neighboring jurisdictions (other communities) that had previously adopted a TRL ordinance, no. (95% CI)**	4.7 (1.5–7.7)	2.2 (1.9–2.6)	.12^e^

Abbreviations: e-cigarette, electronic cigarette; TRL, tobacco retail license.

a Includes the 85 cities and 1 large unincorporated area that had not adopted or strengthened a TRL ordinance to prohibit flavored tobacco products, as of 2019.

b Data sources: 1) the Tobacco Control and Prevention Program ordinance tracking database (unpublished data, 2023), 2) the Los Angeles County Health Survey (unpublished data, LACDPH, 2018), 3) US Census Bureau (9), and 4) the California Tobacco Health Assessment Tool (10).

c Kruskal–Wallis test.

d Fisher exact test.

e Satterthwaite *t* test.

From January 2019 through June 2022, 20 cities and the County of Los Angeles government (responsible for the county’s unincorporated area) adopted a flavored tobacco ban ordinance (Figure); all had previously adopted a tobacco-related ordinance.

**Figure:**
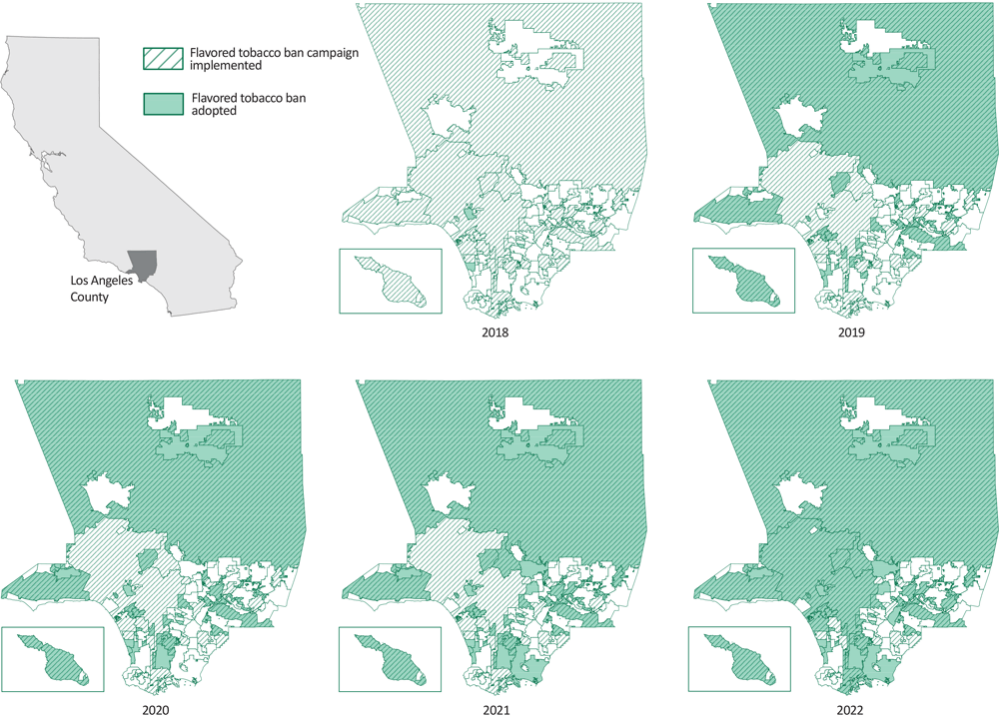
Adoption of local ordinances to restrict the sale of flavored tobacco products in Los Angeles County, 2018–2022. Map for 2018 shows jurisdictions that implemented flavored tobacco ban campaigns from 2019 through 2022. Inset shows Catalina Island. Source: Los Angeles County Department of Public Health.

The likelihood of adopting a flavored tobacco ban ordinance increased by 6% (95% CI, 3%–9%) for every increase in the number of neighboring jurisdictions with a pre-2019 tobacco retail license ordinance (*P* < .001). Eleven of 20 jurisdictions (55.0%) with a flavored tobacco ban campaign adopted a flavored tobacco ban ordinance, while only 10 of 66 jurisdictions (15.2%) without a campaign adopted an ordinance (Table 2). The corresponding crude risk ratio for this finding was 3.6 (95% CI, 1.8–7.3; *P* < .001). After controlling for previous multiunit housing ordinances, other concurrent tobacco-related campaigns, and geographic region, the adoption of a flavored tobacco ban ordinance was 2.2 times more likely in jurisdictions with a flavored tobacco ban campaign than in jurisdictions without a campaign (adjusted risk ratio = 2.2; 95% CI, 1.0–5.0; *P* = .05). Other jurisdictional attributes associated with adoption of a flavored tobacco ban ordinance were previous adoption of tobacco-related ordinances, a larger population size (>100,000 people), and geographic region (Table 2).

**Table 2:** Association Between Jurisdictional Attributes and the Adoption of Local Ordinances to Restrict or Ban the Sale of Flavored Tobacco Products After 2019 in 86 Jurisdictions, Los Angeles County

Attribute	No./total (%) of jurisdictions^a^ adopting a Flavored Tobacco Ban		Crude RR^b^ (95% CI) [*P* value]	Adjusted RR^b^ (95% CI) [*P* value]
	Jurisdictions with attribute	Jurisdictions without attribute		
Implemented a flavored tobacco ban campaign	11/20 (55.0)	10/66 (15.2)	3.6 (1.8–7.3) [<.001]	2.2 (1.0–5.0) [.05]^c^
Previous adoption of tobacco-related ordinance for outdoor areas	21/65 (32.3)	0/21 (0)	Does not converge^d^	Does not converge^d^
Previous adoption of tobacco-related ordinance for multiunit housing	8/12 (66.7)	13/74 (17.6)	3.8 (2.0–7.2) [<.001]	3.2 (1.7–6.2) [.003]^e^
Previous adoption of TRL ordinance	17/43 (39.5)	4/43 (9.3)	4.3 (1.6–11.6) [.005]	3.7 (1.4–10.9) [.01]^e^
Previous adoption of any tobacco-related ordinance	21/67 (31.3)	0/19(0)	Does not converge^d^	Does not converge^d^
>15% of Population living below the federal poverty level	7/17 (41.2)	14/69 (20.3)	2.0 (1.0–4.2)[.06]	1.5 (0.7–3.0) [.31]^f^
>50% of Population aged 21–59 years	19/65 (29.2)	2/21 (9.5)	3.1 (0.8–12.1) [.11]	1.6 (0.4–6.6) [.52]^g^
Population size >100,000	8/16 (50.0)	13/70 (18.6)	2.7 (1.3–5.4) [.005]	^—h^
**Geographic region**				
Central Los Angeles	2/2 (100.0)	19/84 (22.6)	4.4 (3.0–6.6) [<.001]	^—h^
Northwest/Antelope Valley	2/4 (50.0)	19/82 (23.2)	2.2 (0.8–6.2) [.15]	^—h^
San Gabriel Valley	5/28 (17.9)	16/58 (27.6)	0.6 (0.3–1.6) [.34]	^—h^
San Fernando Valley	5/7 (71.4)	16/79 (20.3)	3.5 (1.9–6.7) [<.001]	^—h^
Pomona Valley	2/4 (50.0)	19/82 (23.2)	2.2 (0.8–6.2) [.02]	^—h^
Southeast	5/22 (22.7)	16/64 (25.0)	0.9 (0.4–2.2) [.83]	^—h^
Harbor	4/7 (57.1)	17/79 (21.5)	2.7 (1.2–5.7) [.01]	^—h^
South Bay	4/15 (26.7)	17/71 (23.9)	1.1 (0.4–2.8) [.82]	^—h^
Westside	3/4 (75.0)	18/82 (22.0)	3.4 (1.7–6.9) [<.001]	^—h^
Santa Monica Mountains	2/6 (33.3)	19/80 (23.8)	1.4 (0.4–4.7) [.58]	^—h^

Abbreviations: RR, risk ratio; TRL, tobacco retail license.

a Jurisdictions include the 85 cities and 1 large unincorporated area that had not adopted or strengthened a TRL ordinance to prohibit flavored tobacco products, as of 2019.

b Estimates were obtained by using a modified Poisson regression analysis.

c Adjusted for a previously adopted tobacco-related ordinance for multiunit housing; any other concurrent tobacco-related campaign; and the geographic regions Westside and San Fernando Valley. Similar point estimates were observed when the analysis was adjusted for previously adopted TRL ordinance instead of a previously adopted tobacco-related ordinance for multiunit housing.

d Could not estimate RRs because values in cells were too small; none of the jurisdictions without the attribute adopted a flavored tobacco ban.

e Adjusted for population size.

f Adjusted for population size and proportion of the population aged 21 to 59 years.

g Adjusted for population size, the proportion of the population living below the federal poverty level, and adoption of a previous TRL ordinance.

h No adjustment was needed.

## Discussion

Our study found that jurisdictions that used a community engagement approach (ie, a flavored tobacco ban campaign) were approximately 2 times more likely to adopt a flavored tobacco ban ordinance than jurisdictions where such an approach was not used, after controlling for confounders. This finding affirms the value of using this type of community engagement approach to drive tobacco control at the local level. The flavored tobacco ban campaigns involved the engagement of community partners, city residents, and coalitions to capture the diverse perspectives that are typically required to encourage local government to act. We examined other jurisdictional attributes that may affect the adoption of flavored tobacco bans and discovered that population size, geographic region, number of neighboring communities with a previous tobacco retail license ordinance, and previous experience with adopting tobacco-related ordinances were factors associated with adoption of flavored tobacco ban ordinances.

Similar to other studies that examined the effect of geographic location on policy adoption (12), geographic region or proximity to other communities with a tobacco retail license ordinance resulted in a higher likelihood of a jurisdiction adopting a flavored tobacco ban ordinance. This observation, in part, may be explained by the policy diffusion phenomenon, which occurs when the likelihood of ordinance adoption in one jurisdiction affects the adoption of a similar ordinance in neighboring jurisdictions (13). Data on geographic patterns may be valuable for informing future tobacco control campaigns.

Our study has some limitations. First, findings were conscribed by the existing data sources and the context of the political and cultural environment in California and may not be generalizable to other areas of the US. For example, California passed a flavored tobacco ban in 2020 (14), which went into effect in 2022; this state flavored tobacco ban may have affected the passage of local flavored tobacco bans. Second, selection bias likely affected the study’s observations on the effects of the flavored tobacco ban campaign on adoption of a flavored tobacco ban ordinance. Jurisdictions that had an active campaign were more likely to have previously adopted a tobacco-related ordinance (especially for multiunit housing), and thus, had experience with the ordinance adoption process. We did, however, adjust for this confounder in our analyses.

In Los Angeles County, flavored tobacco bans are becoming more popular and are used by local jurisdictions as a key driver of tobacco control to help reduce the adverse health effects of vaping and use of other flavored tobacco products. A community engagement approach can successfully drive the passage of flavored tobacco ban ordinances, especially in larger populations and in communities that have previously enacted tobacco-related ordinances.
